# Split-Inteins for Simultaneous, site-specific conjugation of Quantum Dots to multiple protein targets *In vivo*

**DOI:** 10.1186/1477-3155-9-37

**Published:** 2011-09-15

**Authors:** Anna Charalambous, Ioanna Antoniades, Neophytos Christodoulou, Paris A Skourides

**Affiliations:** 1 Department of Biological Sciences, University of Cyprus, P.O. Box 20537 1678 Nicosia, Cyprus

## Abstract

**Background:**

Proteins labelled with Quantum Dots (QDs) can be imaged over long periods of time with ultrahigh spatial and temporal resolution, yielding important information on the spatiotemporal dynamics of proteins within live cells or *in vivo*. However one of the major problems regarding the use of QDs for biological imaging is the difficulty of targeting QDs onto proteins. We have recently developed a DnaE split intein-based method to conjugate Quantum Dots (QDs) to the C-terminus of target proteins *in vivo*. In this study, we expand this approach to achieve site-specific conjugation of QDs to two or more proteins simultaneously with spectrally distinguishable QDs for multiparameter imaging of cellular functions.

**Results:**

Using the DnaE split intein we target QDs to the C-terminus of paxillin and show that paxillin-QD conjugates become localized at focal adhesions allowing imaging of the formation and dissolution of these complexes. We go on to utilize a different split intein, namely Ssp DnaB mini-intein, to demonstrate N-terminal protein tagging with QDs. Combination of these two intein systems allowed us to simultaneously target two distinct proteins with spectrally distinguishable QDs, *in vivo*, without any cross talk between the two intein systems.

**Conclusions:**

Multiple target labeling is a unique feature of the intein based methodology which sets it apart from existing tagging methodologies in that, given the large number of characterized split inteins, the number of individual targets that can be simultaneously tagged is only limited by the number of QDs that can be spectrally distinguished within the cell. Therefore, the intein-mediated approach for simultaneous, *in vivo*, site-specific (N- and C-terminus) conjugation of Quantum Dots to multiple protein targets opens up new possibilities for bioimaging applications and offers an effective system to target QDs and other nanostructures to intracellular compartments as well as specific molecular complexes.

## Background

Visualizing protein localization, activity-dependent translocation and protein-protein interactions *in vivo*, in real time has become vital for unraveling the complexity and dynamics of biological interactions [1,2]. Organic fluorophores have been widely used for these purposes but are subject to various limitations, most notably a lack of photostability and relatively low emission intensity, limiting study of long and short term dynamics respectively, especially when imaging takes place *in vivo *and in highly auto-fluorescent embryos [3]. QDs, such as CdSe-ZnS core-shell nanoparticles, are inorganic fluorophores that circumvent these limitations due to their superior optical properties and are thus a promising alternative bioimaging tool. In contrast to organic fluorophores, QDs act as robust, broadly tunable manometers that can be excited by a single light source, offer extremely high fluorescence intensity, wide excitation spectra, narrow and tunable emission spectra, large stokes shift and resistance to photobleaching [4-9].

However QDs have a number of limitations which need to be resolved before their full potential can be realized including i) lack of versatile techniques for selective and site-specific targeting of QDs to biomolecules within specific cell compartments or within molecular complexes *in vivo *(ii) lack of QDs that can be targeted to biomolecules with controllable stoichiometry (iii) lack of compact QDs with small hydrodynamic diameters, close to those of biological macromolecules (iv) lack of methodologies for the efficient delivery of QDs into cells [9,10]. Although some of the above issues are gradually being resolved, site specific targeting of QDs to proteins *in vivo*, still remains a major problem [11,12]. One promising approach is based on the use of polyhistidine peptides (His-tags) fused to proteins of interest. His-tags can bind with high affinity and specificity to bivalent metal atoms such as Ni^2+ ^or Zn^2+ ^and can therefore efficiently assemble on the QD surface with a well-defined orientation [13]. Another approach exploits the highly specific yet non-covalent interaction between the bacterial streptavidin protein and the small molecule vitamin biotin. QDs conjugated to streptavidin can bind with high affinity and specificity to proteins biotinylated under physiological conditions [14]. Furthermore, the use of HaloTag proteins, which are haloalkane dehalogenase bacterial proteins that have been mutated to readily form a covalent bond with chloroalkanes has also been explored [15]. Because chloroalkanes are very rare functional groups in biology, one can label a HaloTag fusion protein with QDs that display chloroalkane groups.

Even though these strategies afford stable QD-protein conjugates capable of withstanding complex biological environments for prolonged periods of time without significant dissociation, they are restrictive in that they do not allow labelling of different proteins simultaneously for multiparameter imaging of cellular functions. To address this challenge, we decided to take advantage of an intein-mediated ligation system. Inteins are polypeptide sequences that are able to self-excise during a process termed protein splicing, rejoining the two flanking extein sequences by a native peptide bond [16-21]. Molecular mechanisms of protein splicing have been studied and they involve N → S (or →O) acyl shift at the splice sites [18,22,23], formation of a branched intermediate [24,25] and cyclization of an invariant Asn residue at the C-terminus of the intein to form succinimide [26], leading to excision of the intein and ligation of the exteins. Inteins have been widely used for *in vitro *protein semi-synthesis [20,27], segmental isotopic labelling [28], QD nanosensor synthesis [29-31]*in vivo *protein cyclization [32,33] and *in vivo *conjugation of QDs to biomolecular targets [34]. Nearly 200 intein and intein-like sequences have been found in a wide variety of host proteins and in microorganisms belonging to bacteria, archaea and eukaryotes [35]. Inteins share only low levels of sequence similarity but they share striking similarities in structure, reaction mechanism and evolution [36]. It is thought that inteins first originated with just the splicing domain and then acquired the endonuclease domain, with the latter conferring genetic mobility to the intein [35]. During intein evolution however, some inteins lost sequence continuity, such as the DnaE split intein, and as a result they exist in two fragments capable of protein trans splicing [37].

**Figure 1:**
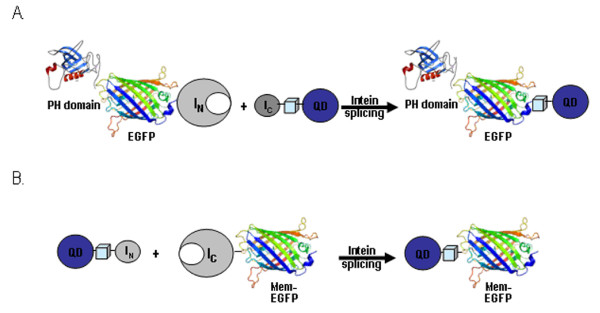
***In vivo *conjugation of QDs to the C- or N-terminus of target proteins via intein mediated protein splicing**. **(A) **Schematic representation of site-specific Ssp DnaE split intein-mediated conjugation of QDs to the C-terminus of the PH domain of Akt. **(B) **Schematic representation of site specific Ssp DnaB mini intein-mediated conjugation of QDs to the N-terminus of mem-EGFP.

We have recently used the DnaE split intein to site-specifically conjugate QDs to the C-terminus of the PH domain of Akt and Btk, *in vivo *(Figure 1A )[34]. We have now utilized a new split intein to allow conjugation of QDs to the N-terminus of target proteins. This expands the possibilities of the intein-based system allowing for the first time *in vivo *site specific conjugation of QDs and other nanostructures to the N terminus of target proteins. We selected the Ssp DnaB mini-intein, to achieve N-terminal protein QD labelling, given that the N-terminal part was small enough to be synthetically produced and shown to be capable of trans-splicing and protein modification [38,39]. This mini-intein lost its endonuclease domain during evolution and currently consists of just the 130aa protein splicing domain plus a 24aa linker sequence in place of the endonuclease domain [35,40]. Recent work by Sun W. et al. demonstrated that the Ssp DnaB mini-intein remained proficient in protein trans-splicing when artificially split in the loop region between the β-strands, β2 and β3, producing an N-terminal part of 11 aa and a C-terminal part of 143aa (Figure 1B**) **[41].

We go on to show that inteins can be used to target QDs to specific molecular complexes within living cells and embryos. Specifically through the targeting of QDs to the C-terminus of paxillin, we generated a full length protein-QD complex. Paxillin-QD conjugates localized efficiently to focal adhesion complexes within the cells of the developing embryo. Imaging of these complexes in real time revealed that QDs would associate with newly formed focal adhesions and would be released once the complexes were disassembled. Finally, split intein based QD conjugation may be extended to simultaneous and multiple protein tagging as long as functionally orthogonal split inteins are used, in order to prevent undesired side products due to cross-reactivity [42]. Through the combination of the C and N terminal intein systems we were able for the first time to simultaneously target two distinct proteins with spectrally resolvable QDs *in vivo*. This is to our knowledge the only methodology that will allow conjugation of multiple targets with QDs without cross reactivity and should serve as an important addition to existing labeling methods.

## Results and Discussion

### Quantum Dots targeted to Focal Adhesion Complexes following *in vivo*, intein-mediated conjugation to the C-terminus of paxillin

**Figure 2:**
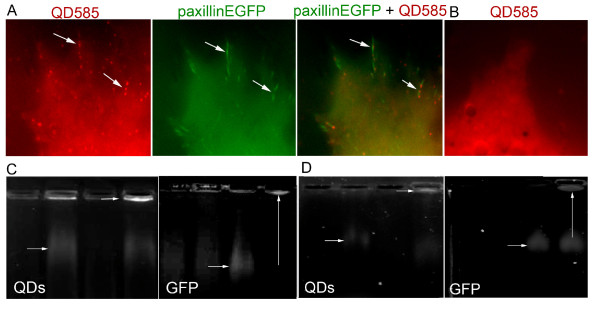
**In vivo conjugation of QDs to the C-terminus of Paxillin-EGFP via intein mediated protein splicing.** Co-localization of QDot_585_ with Paxillin-EGFP on focal-adhesions of mesodermal cells during migration. Stage 2 *Xenopus* embryos were injected with (A) probe (DnaE I_C_-QDot_585_)(in red) and RNA encoding Paxillin-EGFP-DnaE I_N_ (in green) or (B) probe (DnaE I_C_-QDot_585_) alone. Fluorescence images of animal cap cells dissociated from stage 8 *Xenopus* embryos, induced with activin, and plated onto fibronectin coated slides. **(A)** Yellow shows overlap between red QDot_585_ and green EGFP indicating successful QD-protein conjugation in vivo. **(B)** In the absence of Paxillin-EGFP, QDs do not target focal adhesions but remain diffusely localized in the cytosol. **(C &D)** Biochemical characterization of protein-QD conjugates. *Xenopus* embryos were injected as follows, **C**: i) Uninjected ii) DnaE I_C_-QDot_585_ ii) Paxillin-EGFP-DnaE IN RNA iii) DnaE IC-QDot_585_ + Paxillin-EGFP-DnaE IN RNA, **D**: i) Uninjected ii) QDot_585_-DnaB I_N_ iii) DnaB I_C_-memEGFP RNA iv) QDot_585_-DnaB I_N_ + DnaB I_C_-memEGFP RNA, lysed at stage 10 and loaded onto a 0.5% agarose gel in this order, from left to right. QDot_585_ were visualized with the ethidium bromide emission filter under UV excitation and EGFP was imaged with a band pass 500/50 filter set on *UVP iBox*Imaging System. The ligation products appear as a single band under the GFP and QD filters, only in lysates of *Xenopus* embryos injected with RNA + QD probe (vertical white arrows). Bands corresponding to Paxillin-EGFP and memEGFP proteins not conjugated to QDs are detectable under the GFP filter, in lysates of *Xenopus* embryos injected with RNA only and QD probes + RNA, but not QDs only (horizontal arrows). Bands corresponding to QD probes are detectable under the QD filter, in lysates of *Xenopus* embryos injected with the QD probes only or the QD probes + RNA, but not RNA only, (horizontal arrows).

We have recently used intein based conjugation to covalently conjugate QDs to the C-terminus of the Plekstrin homology domain of Akt. Using this methodology we were able to site-specifically tag a protein domain with QDs *in vivo *for the first time, effectively generating QD biosensors that could respond to PI3K activation by translocating to the cell membrane [34]. We now wanted to examine whether this methodology could be used i) to tag a full length protein and more importantly ii) to target QDs to specific molecular complexes within the cell. We decided to target paxillin, a focal-adhesion associated protein implicated in the regulation of actin cytoskeletal organization and cell motility [43]. To investigate whether we could target QDs to focal adhesion complexes via paxillin *in vivo*, we injected both blastomeres of 2-cell stage *Xenopus *embryos with the probe (DnaE I_C_-QDot_585_) and RNA encoding the target protein (in this case, Paxillin-EGFP-DnaE I_N_). The presence of EGFP on the paxillin was required as it would allow us to monitor and compare the distribution of the QDs vs paxillin. Embryos were allowed to develop to stage 8, at which point animal cap cells were dissociated, induced with activin, plated onto fibronectin coated slides and observed by time-lapse microscopy. We first examined the localization of Paxillin-EGFP and found that, as previously reported, it localized at focal-adhesions, especially at the filopodia and lamelipodia, generated by mesodermal cells during migration on fibronectin substrates (Figure 2A**) **[44]. Furthermore, QDs translocated to focal adhesions in cells derived from embryos injected with both DnaE I_C_-QDot_585 _and RNA, where they colocalized with Paxillin-EGFP (Figure 2A). On the other hand, in cells that did not express the Paxillin-EGFP-DnaE I_N_, QDs remained in the cytosol (Figure 2B).

**Figure 3:**
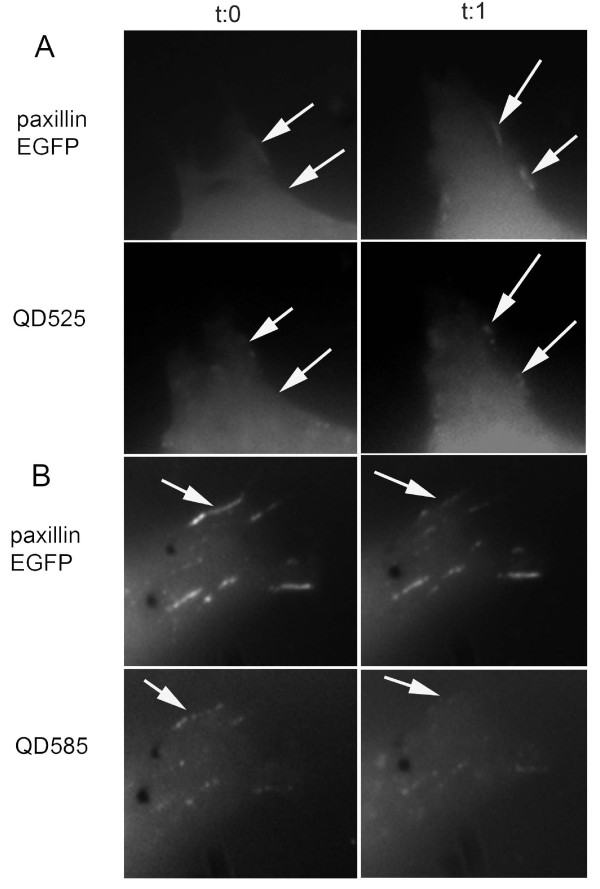
**Paxillin-QD conjugates associate with newly formed focal adhesion complexes and are released once the complexes are disassembled**. *Xenopus *embryos were injected at the 2-cell stage with the probe (DnaE I_C_-QDot_525 or 585_) and RNA encoding Paxillin-EGFP-DnaE I_N_. Animal cap cells were dissociated from stage 8 *Xenopus *embryos, induced with activin, and plated onto fibronectin coated slides. **(A) **Time lapse images (time-interval: 30 sec) show paxillin-QD conjugates associating with newly formed focal adhesion complexes at the filopodia and lamellipodia of mesodermal cells during their migration on fibronectin substrates (see arrows). **(B)** Time lapse images (time-interval: 10 sec) show paxillin-QD conjugates being released from focal adhesion complexes as they disassemble during migration of mesodermal cells on fibronectin substrates (see arrows).

To confirm formation of QD-protein conjugates we used a biochemical approach. *Xenopus *embryos were injected as follows: i) Uninjected ii) DnaE I_C_-QDot_585 _ii) RNA encoding Paxillin-EGFP-DnaE I_N_, iii) DnaE I_C_-QDot_585 _+ RNA encoding Paxillin-EGFP-DnaE I_N_. Embryos were lysed when they reached stage 10 and loaded onto an agarose gel. QDot_585 _were visualized with the ethidium bromide emission filter under UV excitation and EGFP was imaged with a band pass 500/50 filter set on *UVP iBox *Imaging System. As shown in Figure 2C a smeary band of the expected molecular weight for the Paxillin-EGFP appeared in lysates of *Xenopus *embryos injected with the RNA encoding the corresponding target protein. This band could not be detected in lysates of uninjected *Xenopus *embryos or *Xenopus *embryos injected with the probe (I_C _peptide conjugated QD_585_) only. Higher MW bands corresponding to the semi-synthetic products appeared only in lysates of *Xenopus *embryos injected with both the RNA encoding the target protein (Paxillin-EGFP) and the probe (I_C _peptide conjugated QD_585_). Importantly, this new band overlaps with the QD signal. Commercially available streptavidin-coated QDs bear 4-10 streptavidin molecules (53 kD each)/QD giving 16-40 biotin binding sites implying 16-40 conjugated Paxillin-EGFP protein molecules per QD, resulting in a significant increase in size that results in trapping of the conjugates in the gel wells and preventing their migration. Video microscopy revealed that focal adhesion formation and disassembly is very rapid in these highly migratory cells. In addition and as shown in time lapse images, QDs would associate with newly formed focal adhesion complexes (Figure 3A) and would be released once the complexes were disassembled (Figure 3B).

**Figure 4:**
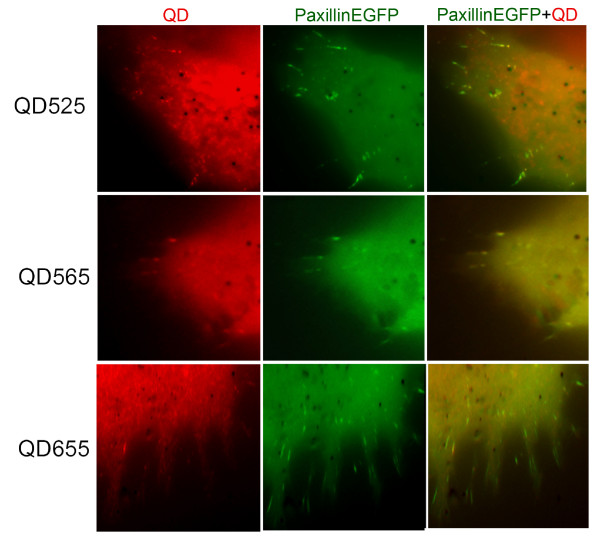
**Increased QD size imposes constraints on the translocation efficiency of Paxillin-EGFP-QD conjugates to the focal adhesion complexes**. Co-localization of QDots_525_, QDots_565 _and QDots_655 _with Paxillin-EGFP on focal adhesion complexes. Note that unlike QDot_525_, the QDot_655 _are not recruited as effectively to the focal adhesion complexes.

We repeated the above described experiment using commercially available QDs from Invitrogen (15-20 nm in diameter) from all the emission wavelengths (525, 565, 585, 655) coupled to streptavidin. Conjugates of paxillin-EGFP with QDs from all the emission wavelengths tested were successfully targeted to the focal adhesions. However, there was a definitive size dependence in their ability to target focal adhesions, with longer wavelength emitting QDs showing a diminished capacity to do so (Figure 4). These results emphasize the need for the generation of biocompatible and colloidally stable long wavelength QDs with smaller effective hydrodynamic radii.

### Quantum Dots targeted to the cell membrane, following *in vivo *intein-mediated conjugation to the N-terminus of a membrane targeted variant of EGFP (memEGFP)

Although intein-based C-terminal conjugation of QDs to proteins is a valuable tool, it is often necessary to tag a protein at the N-terminus in order to achieve a functional conjugate. Thus, we wanted to implement an intein based strategy that would enable site-specific N-terminal conjugation *in vivo*, to complement the C-terminal tagging system we have already described [34]. In addition we wanted to test a shorter synthetic peptide that would make this approach more affordable as well as easy. The value of using short synthetic intein peptides capable of trans-splicing was initially demonstrated for C-terminus-specific modifications of recombinant proteins using artificially split Npu DnaE [45] and Ssp GyrB inteins [42] and more recently for N-terminus-specific modifications using Ssp DnaB mini-intein [41]. By taking advantage of the latter we implemented the strategy shown in Figure 1B. We went on to examine whether this approach could be used successfully for *in vivo *conjugation of QDs to the N-terminus of target proteins using a membrane-targeted variant of EGFP as a target. This construct, generated by the genetic fusion of the enhanced GFP to the farnesylation sequence of p21(Ras) (memEGFP) was selected due to its ability to constitutively localize to the cell membrane as it would provide clear visual confirmation of successful conjugation in the intact embryo [46]. In addition, it is a good example of a target protein that cannot be QD-tagged at the C terminus as that would interfere with the membrane tethering function of the farnesylated residues and would lead to elimination of membrane anchoring.

**Figure 5:**
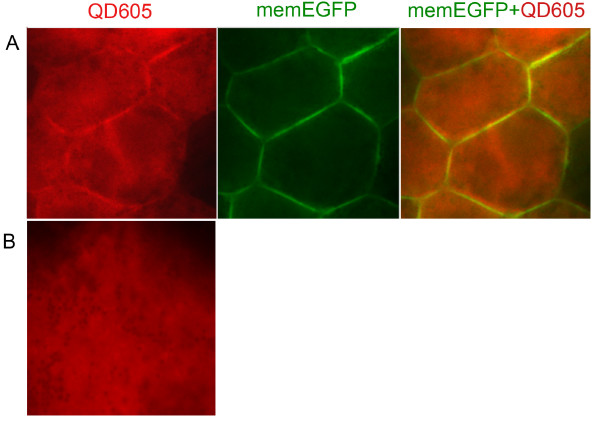
***In vivo *conjugation of QDs to the N-terminus of mem-EGFP via intein mediated protein splicing**. **(A) **Co-localization of QDot_605 _with mem-EGFP on the cell membrane. Fluorescence images of stage 10 *Xenopus *embryos microinjected with the probe (QDot_605_-DnaB I_N_) shown in red, in one blastomere at the two-cell stage, and then injected with RNA encoding the target protein (DnaB I_C_-memEGFP) shown in green, in three of four blastomeres. Yellow shows the overlap between red QDot_605 _and green EGFP indicating successful QD-protein conjugation in a live embryo. **(B) **In embryos injected with the probe (QDot_605_-DnaB I_N_) alone, in the absence of RNA encoding the target protein (DnaB I_C_-memEGFP), QDs do not target the cell membrane but remain diffusely localized in the cytosol.

**Figure 6:**
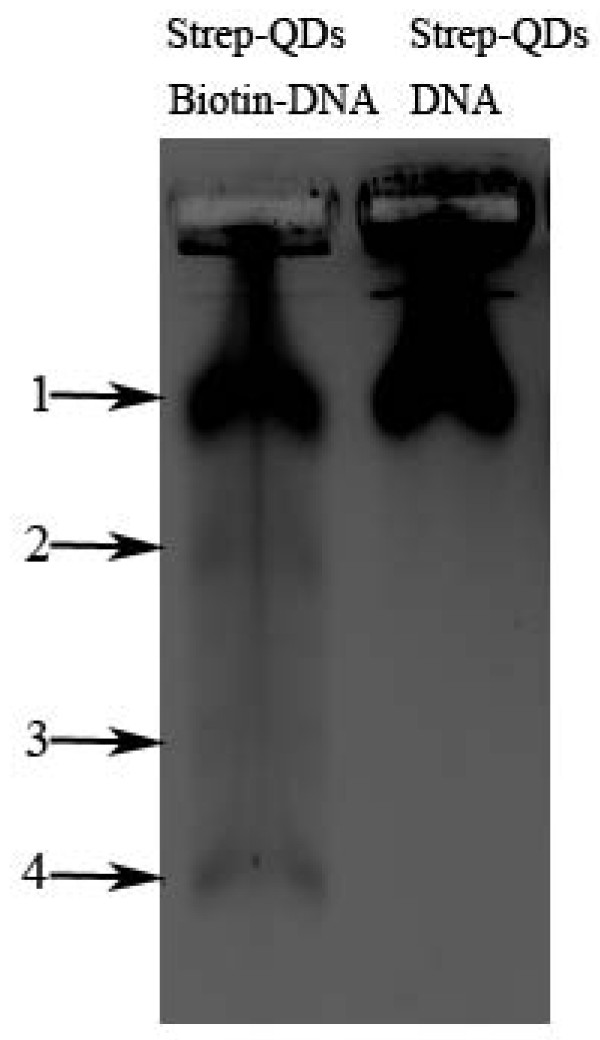
**Evaluation of commercially available streptavidin coated QDs**. Commercially available streptavidin coated QDot_605 _(from Invitrogen) were incubated with biotinylated DNA (lane 1) and non biotinylated DNA (lane 2) at a molar ratio of 1:100, for 30 minutes at room temperature. Following the conjugation reaction the DNA-QD mixtures were run on a 1% agarose gel to assess the percentage of QDs capable of efficient biotin-streptavidin conjugation. QDot_605 _were imaged using the ethidium bromide filter set on the *UVP iBox*Imaging System. As shown, the QDs used in our experiments exhibit great variability in terms of their biotin binding ability (see arrows). Arrow 1 indicates QDs that are unable to bind biotin.

To demonstrate *in vivo *N-terminal labelling of memEGFP with QDs, we injected both blastomeres of two-cell stage *Xenopus *embryos with the probe (QDot_605_-DnaB I_N_) and with RNA encoding the target protein (in this case, DnaB I_C_-memEGFP). As shown in Figure 5A, QDs translocated to the cell membrane in cells derived from the embryo injected with both QDot_605_-DnaB I_N _and RNA, where they colocalized with memEGFP. On the other hand, in cells that do not express the DnaB I_C_-memEGFP, QDs were not targeted to the membrane but remained in the cytosol (Figure 5B). Despite the fact that most QDs colocalize with the target protein to the plasma membrane, a significant amount of QDs remain in the cytosol. This is due to the fact that the initial streptavidin QD solution contains a mixture of streptavidin-conjugated and unconjugated QDs as shown in Figure 6, as well as due to gradual loss of both the intein peptide and the target protein from the QD surface, as a result of proteolytic degradation, as discussed in the Conclusions section. This problem will be significantly ameliorated when QDs with more stable surface modifications become commercially available.

In order to confirm conjugation of QDs to the N-terminus of memEGFP biochemically, we prepared lysates from injected embryos, which were run on an agarose gel, in a similar fashion to what has already been described above for the C-terminal conjugation of QDs to paxillin. As shown in Figure 2D, conjugation of QDs to the N-terminus of memEGFP was successful leading, to a higher molecular weight product, absent from the QD only lane.

### Ssp DnaE and DnaB inteins do not cross splice and therefore facilitate simultaneous targeting of Quantum Dots to two different proteins *in vivo*

**Figure 7:**
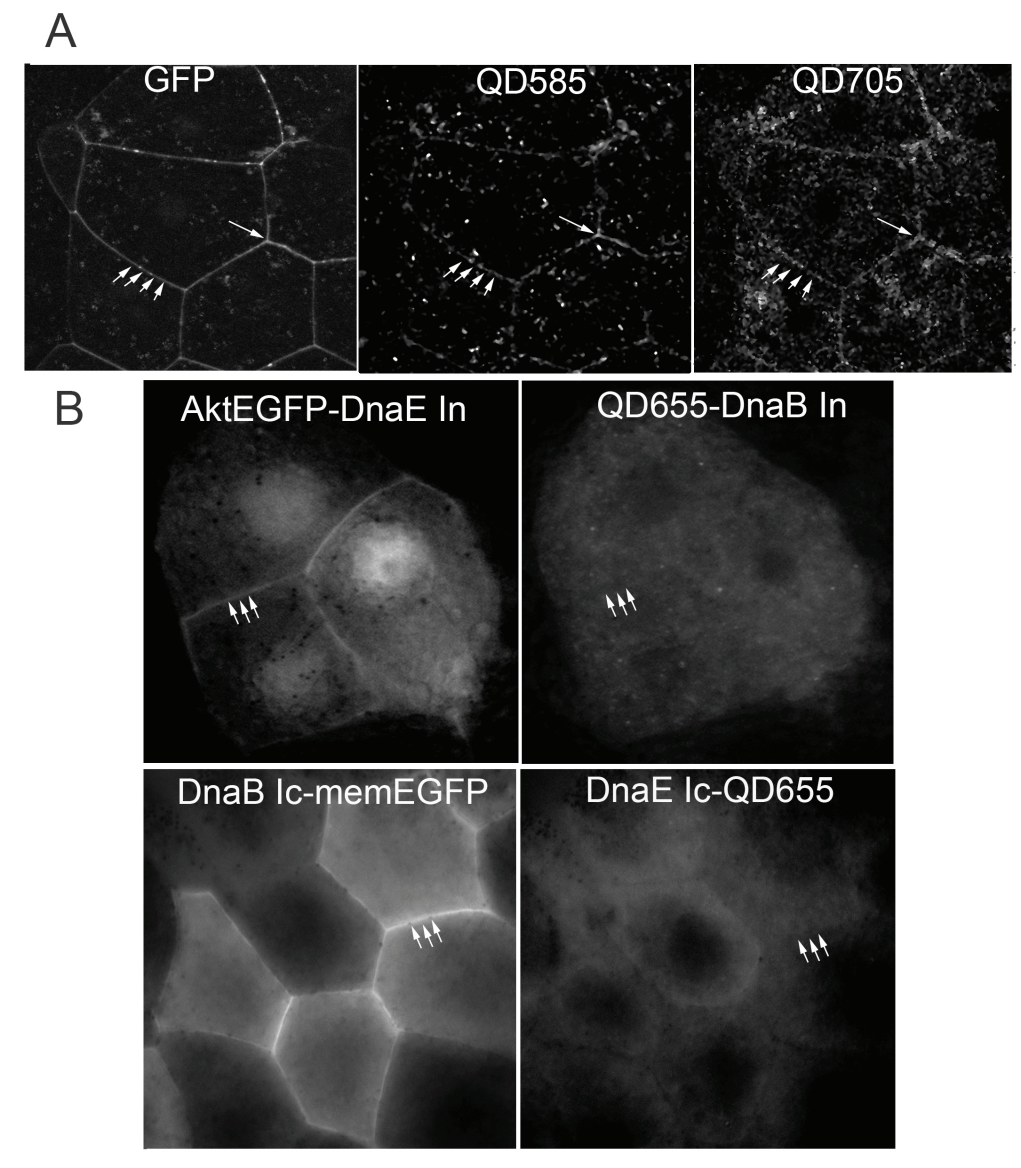
**Simultaneous targeting of QDs to two different proteins via Ssp DnaE and Ssp DnaB intein mediated splicing without cross reactivity**. Fluorescence images of stage 10 *Xenopus *embryos injected with **(A) **the probes QDot_585_-DnaB I_N _and DnaE I_C_-QDot_705 _and the corresponding RNAs encoding DnaB I_C_-memEGFP and Akt-EGFP-DnaE I_N_, **(B) **the probe QDot_655_-DnaB I_N _and RNA encoding Akt-EGFP-DnaE I_N _or the probe DnaE I_C_-QDot_655 _and RNA encoding DnaB I_C_-memEGFP. Both QDot585 and QDot705 translocated to the cell membrane in cells derived from the embryo injected with the complementary probes where they colocalized with memEGFP and Akt-EGFP. In contrast, Akt-EGFP and mem-EGFP clearly target to the cell membrane whereas the non-complementary probes, remain diffuse in the cytoplasm, implying that the two inteins do not cross react.

Several naturally occurring and artificially split inteins have been examined for their orthogonality and it was found that inteins can cross-splice when sharing a high degree of sequence identity and similarity. In fact it has been shown that the natural DnaE split inteins from Nostoc Punctiforme and Synechocystis sp. PCC 6803 cross-splice [47] as do the DnaE split inteins from three other cyanobacteria (Nostoc sp.PCC7120, Oscillatoria Limnetica and Thermosynechococcus vulcanus) [48]. Given that the naturally occurring Ssp DnaE split intein and the artificially split mini intein, Ssp DnaB, do not share any sequence similarity as indicated by a protein-protein BLAST and can afford effective conjugation of QDs to the C- and N-terminus of target proteins respectively we decided to exploit this combination for QD-targeting to multiple proteins *in vivo*, simultaneously. To demonstrate that memEGFP and Akt-EGFP fusion proteins can be simultaneously and specifically targeted by spectrally resolvable QDs, without cross reactivity we performed *in vivo *injections with a mixture of complementary QD-intein peptide probes and target protein RNAs. More specifically we injected both blastomeres of two-cell stage *Xenopus *embryos with the probes QDot_585_-DnaB I_N _and DnaE I_C_-QDot_705 _and the corresponding RNAs encoding DnaB I_C_-memEGFP and Akt-EGFP-DnaE I_N_. As shown in Figure 7A, both QDot585 and QDot705 translocated to the cell membrane in cells derived from the embryo injected with the complementary probes where they colocalized with memEGFP and Akt-EGFP. We predicted that the N-terminus of the DnaE intein would not react with the C-terminus of the DnaB intein and vice versa, as the specific interactions that facilitate the splicing reaction, notably recognition of the complementary N- or C-intein and consequent non-covalent association for formation of an active-intein intermediate, could not be formed given that there is no sequence similarity. To examine if cross splicing between Ssp DnaE and Ssp DnaB inteins occurs we injected both blastomeres of two-cell stage *Xenopus *embryos with the probe QDot_655_-DnaB I_N _and RNA encoding Akt-EGFP-DnaE I_N_. As shown in Figure 7B, Akt-EGFP clearly target to the cell membrane whereas QDot_655 _remain diffuse in the cytoplasm. Clearly, Akt-EGFP-QD conjugates do not form, implying that the two inteins cannot cross splice. Similar results were obtained when we examined the reverse combination, that is when we injected two-cell stage *Xenopus *embryos with the probe DnaE I_C_-QDot_655 _and RNA encoding DnaB I_C_-memEGFP (Figure 7B).

This experiment thus demonstrates that intein-mediated trans splicing facilitates simultaneous and specific tagging of two protein targets within the same embryo with spectrally resolvable QDs without cross splicing. Given the large number of orthogonal inteins it is possible that more than two targets can be simultaneously tagged with different QDs or different nanostructures.

## Conclusions

Herein, we describe an intein-based system for conjugation of QDs to target proteins *in vivo*. This approach has several advantages over existing methodologies that make it truly unique, including i) site-specificity (N- or C-terminus), ii) low-intrinsic reactivity towards endogenous proteins which do not contain the intein motif required for splicing, thus eliminating mis-targeting of the QDs, iii) versatility conferred by the ability to target QDs to a single protein within any cellular compartment or molecular complex and iv) the ability to target spectrally resolvable QDs to multiple protein targets simultaneously without cross reactivity.

We have previously shown site-specific conjugation of QDs to the C-terminus of target proteins by using the naturally-split DnaE intein [34]. However, C-terminal protein labelling with QDs can in some cases, interfere with protein localization and/or biological function, as can C-terminal fusion of fluorescent proteins [49-51]. This is due to interference with protein sorting or targeting signals located at the C-terminus of proteins, such as two common ER retrieval signals, the dilysine motif and the tetrapeptide KDEL, as well as the type 1 peroxisomal targeting signal peptide SKL [50]. A C-terminal tag or marker could also disrupt signals for the incorporation of lipid anchors. For example, many members of the Ras superfamily carry sequences that signal the attachment of lipid anchors at their C-termini [51]. A class of plasma membrane proteins, including cell adhesion molecules or receptors have a glycosylphosphatidylinositol (GPI) linker [49]. The molecular signals engaging the lipid modification enzyme complexes reside at the C-terminus of these proteins and would definitely be disrupted by the addition of a fluorescent protein or QD. We therefore took advantage of the artificially split Ssp DnaB intein originally described by Sun, W. et al. [41], for site-specific conjugation of QDs to the N-terminus of target proteins. Ssp DnaB intein has been split artificially at a site (S1) proximal to the N- terminal, producing an N-terminal piece of only 11 aa in length and a C-terminal piece of 144 aa in length [41]. This novel artificially split intein is quite useful due to the ease of chemical peptide synthesis and due to the fact that such short peptides are not prone to misfolding. We used the S1 split intein for site-specific conjugation of QDs to the N-terminus of a model target protein in vivo, namely mem-EGFP, and have shown that QD-memEGFP conjugates localize to the cell membrane and can be monitored in real time within the developing *Xenopus *embryo (Figure 5). Thus, the ability to target QDs to the N-terminus of proteins is very helpful for bioimaging studies aiming at determining protein localization and function, given that there are numerous proteins bearing C-terminal post-translational modifications or a C-terminal critical domain whose function would be impeded if a bulky QD was conjugated at the C-terminus.

We have also demonstrated, using this methodology, that Quantum Dots can be targeted via paxillin to focal adhesions, a specific molecular complex, for the first time. Focal Adhesions (FAs) are comprised of α and β integrin heterodimers that form a bridge between the intracellular actin cytoskeleton and the extracellular matrix (ECM) [52]. While the extracellular domain of integrins binds directly to ECM proteins, the cytoplasmic tail is linked to the actin cytoskeleton via signalling and adapter proteins, such as focal adhesion kinase (FAK), vinculin, talin and paxillin [52]. FAs play a crucial role in cell adhesion, spreading and motility by regulating various signal transduction pathways leading to rearrangement of the actin cytoskeleton [53,54]. We have demonstrated that QDs can be efficiently targeted to focal adhesions via paxillin without altering protein localization and/or function. In fact Paxillin-QD conjugates retained full functionality as indicated by their ability to i) translocate to focal adhesions at the cell membrane (Figure 2A) and ii) associate with newly formed focal adhesion complexes and be released once the complexes were disassembled (Figure 3). This is an inherent advantage of QDs over fluorescent proteins since the former are conjugated to target proteins post-translationally and do not therefore interfere with protein folding and tertiary structure.

A useful additional application of this intein-based methodology is the simultaneous and specific conjugation of QDs to multiple proteins targets in vivo. Although fluorescent proteins already provide a straightforward solution to this problem [3]. QD-conjugation methods are attractive complements given the superior optical properties of QDs over fluorescent proteins [55]. Double in vivo labeling becomes possible with our system due to the existence of orthogonal pairs of split inteins that do not cross splice and therefore allow different protein targets to be simultaneously and specifically tagged with spectrally resolvable QDs within the cell. Such orthogonal split-intein combinations include Ssp DnaE and Sce VMA, Ssp DnaB and Sce VMA, Ssp DnaB and Mxe GyrA [42] to mention but a few and now Ssp DnaE and Ssp DnaB. In fact, given the large number of characterized split inteins, the number of individual targets that can be simultaneously tagged is only limited by the number of QDs that can be spectrally distinguished. Moreover, the fact that the trans-splicing reactions proceed with an identical molecular mechanism ensures similar reaction rates for QD-conjugation that would aid the comparison of the properties of the two proteins-otherwise the first protein of interest is already redistributing while the second protein is not yet sufficiently labelled. We have shown in this work that Ssp DnaE and Ssp DnaB inteins do not cross splice and may therefore be used to specifically target spectrally resolvable QDs to different proteins simultaneously in vivo (Figure 7).

Despite the successful conjugation of QDs to both the N and C terminus of target proteins, the current methodology and the materials used have certain limitations that need to be noted. We have observed that a pool of QDs remains in the cytosol, even when the target protein is in excess. This was expected in the case of paxillin, a cytosolic protein occasionally localized to the focal adhesion complexes on the cell membrane, but came as a surprise in the case of memEGFP, a protein expected to be exclusively localized on the cell membrane. An unwanted result of the presence of free QDs in the cytosol was the reduction of signal to noise ratio. These QDs are most likely not conjugated to the target protein due to the following two reasons. Firstly the commercially available solution of Streptavidin-coated QDs used in these experiments, contains both streptavidin-conjugated and free QDs (see Figure 6). This implies that even if the splicing reaction is 100% efficient, a portion of free QDs is still present in the cell. Secondly, in the *Xenopus *model, translation begins after the Midblastula Transition (~12 hours post injection). By that time, a portion of the streptavidin-conjugated QDs may have lost the streptavidin or the intein peptide (due to proteolytic degradation). This, in effect, generates additional free QDs, which will remain in the cytosol, thus reducing the apparent conjugation efficiency. Given that as the embryo develops, the amount of conjugated QDs is progressively reduced and given the target proteins' degradation rate, it is important to note that the time frame for imaging can be quite small. In addition, the presence of free QDs in the cytosol greatly impedes visualization of target proteins that do not localize to a specific organelle or structure in the cell, even early on. These limitations raise the need for i) commercially available QDs capable of retaining their conjugated biomolecule longer and ii) improved methodologies to ensure that the starting material consist of 100% conjugated QDs.

Our present results indicate efficient, covalent and site-specific in vivo-fusion of QDs to either the N- or C- terminus of a target protein within any cellular compartment or molecular complex. This methodology is notable due to its potential diagnostic and therapeutic applications, as it makes the targeting of nanostructures and nanodevices to different intracellular compartments and signalling complexes a viable possibility. Furthermore, this method is unique in that it facilitates QD conjugation to multiple target proteins, as long as orthogonal intein pairs are used. The number of potential applications for double (or multiple) in vivo labelling is quite large. Most obviously, protein localizations of two or more species can be followed simultaneously and protein-protein interactions may be explored using QDs suited for FRET experiments. In conclusion the intein-mediated approach for simultaneous, in vivo, site-specific (N- and C-terminus) conjugation of Quantum Dots to multiple protein targets, should serve as a powerful tool for bioimaging applications.

## Methods

### Embryos and explants

*Xenopus laevis *embryos from induced spawning [56] were staged according to Nieuwkoop and Faber (1967). Operation techniques and buffer (MMR, Ubbels, 1983) have been described [56]. *Xenopus *embryos were fertilized *in vitro *and dejellied using 2% cysteine-HCl, pH 7.8, then maintained in 0.1× Marc's Modified Ringer's (0.1× MMR). Microinjections were performed in 4% Ficoll in 0.33× MMR. The embryos were injected with RNA and QDs conjugated to either the C-terminal part of DnaE Intein (DnaE-I_C_) or the N-terminal part of Ssp DnaB mini-intein (DnaB-I_N_) through a biotin-streptavidin bond, at the 2 and 4-cell stage according to established protocols [57]. After injections the embryos were cultured in 4% Ficoll in 0.33× MMR until stage 8 and then cultured in 0.1× MMR at room temperature. For *in vivo *assays, the embryos were transferred to slides for time lapse movies using Zeiss Axiocam MR3 and the Axiovision software 4.6 to monitor GFP-QD co-localization.

### Electrophoretic evaluation of streptavidin-coated QDs

Commercially available streptavidin coated QDot_605 _(from Invitrogen) were incubated with biotinylated DNA and non biotinylated DNA at a molar ratio of 1:100, for 30 minutes at room temperature. Following the conjugation reaction the DNA-QD mixtures were run on a 1% agarose gel to assess the percentage of QDs capable of efficient biotin-streptavidin conjugation. QDot_605 _were imaged using the ethidium bromide filter set on the *UVP iBox *Imaging System.

### Chemical Synthesis of biotinylated C-terminus DnaE intein peptide (DnaE I_C_-Biotin) and biotinylated N-terminus DnaB mini-intein peptide (Biotin-DnaB I_N_)

The 47 amino acid peptide sequence of the C-terminus DnaE intein peptide (DnaE I_C_-Biotin):

MVKVIGRRSLGVQRIFDIGLPQDHNFLLANGAIAANCFDYKDDDDK(Ahx-Biotin)G

The 11 amino acid peptide sequence of the N-terminus DnaB intein peptide (Biotin-DnaB I_N_):

Biotin-KKK-Ahx-CISGDSLISLA

Biotin was conjugated to a C-terminal Lysine (K) on DnaE I_C _via an Ahx linker (6 carbon inert linker) and to a N-terminal Cysteine (C) on DnaB I_N _via a three lysine linker and Ahx. Both peptides were synthesized on a 0.5 mmol scale on a 4-methylbenzhydrylamine (MBHA) resin according to the in-situ neutralization/HBTU activation protocol for Boc SPPS [58]. In order to put a biotin at the C-terminus of DnaE intein, it was necessary to add an extra amino acid, Lys, at the C-terminus. In order to put a biotin at the N-terminus of DnaB intein, it was necessary to add three extra Lys, at the N-terminus. Lysines serve as a linking point for biotin as well as a spacer between the peptide and biotin. The DnaE I_C _peptide contains a cysteine protected with the NPyS group which was added as the last amino acid in the synthesis. Following chain assembly, global de-protection and cleavage from the support was achieved by treatment with HF containing 4% v/v pcresol, for 1 hour at 0°C. Following removal of the HF, the crude peptide products were precipitated and washed with anhydrous cold Et_2_O before being dissolved in aqueous acetonitrile (50% B) and lyophilized. The crude peptides were purified by preparative HPLC using a linear gradient of 25-45% B over 60 minutes. The purified peptides were characterized as the desired product by ESMS. The lyophilized biotinylated DnaE I_C _peptide was dissolved in 60% DMSO at a concentration of 1 mg/ml. The lyophilized biotinylated DnaB I_N _peptide was dissolved in PBS at a concentration of 1 mg/ml.

### In vitro conjugation of DnaE I_C_-Biotin and Biotin-DnaB I_N _to streptavidin-coated QDs

The biotinylated peptides were diluted to 50 μM and used at 1:1 volume ratio with streptavidin-coated QDs (1 μM) (from Invitrogen or eBiosciences). To allow formation of the biotin-streptavidin bond we incubate at 24°C for 30 min. To remove any excess unbound peptide the conjugate was filtered through microcon centrifugal filter units (YM100) [59].

### Analysis of QD-peptide conjugates

Analysis of QD-peptide conjugation was performed by electrophoresis at 60 V for 4 h at 4°C using a 0.5% agarose gel. No loading buffer was added to the samples before loading. Gels were visualized under the ethidium bromide filter (515-570 nm) with a UVP Imager (data not shown).

Alternatively analysis of QD peptide conjugation was performed by spotting nitrocellulose membranes (Whatman). Biotinylated peptides and peptides that did not contain the biotin modification were spotted on nitrocellulose membrane and blocked in PBS containing 1% BSA for 30 min at room temperature. The nitrocellulose membrane was then soaked in PBS containing streptavidin-coated QDs (1:500 dilution) for 30 min at room temperature. The membrane was washed with PBS-Tween 20 (1%) twice and visualized under the ethidium bromide filter (515-570 nm) with a UVP Imager (data not shown).

### Plasmids and Cloning

All plasmids were constructed using standard molecular biology techniques and they were sequenced to verify correct coding.

pCS2++-Paxillin-EGFP- I_N_

A PCR fragment amplified with F_pax _(5' AAATCGATATGGACGACCTCGAT 3') and R_egfp _(5' CCGAATTCCTTGTACAGCTCGTC 3') encoding paxillin-EGFP, using the pEGFP-N3 plasmid (from Addgene) as template was inserted into the multiple cloning site of the pCS2++ plasmid by restriction enzyme digest with ClaI-EcoRI. A PCR fragment amplified with IGpr61 (AAGGAATTCAAGTTTGCGGAATATTGCCTCAGTTTTGG) and IGpr63 (AAGCTCGAGTTATTTAATTGTCCCAGCG) encoding I_N _with 5 N-terminal extein residues (KFAEY), using the pJJDuet30 plasmid (from Addgene) as template was inserted at the C-terminus of Paxillin-EGFP on pCS2++ between the EcoRI-XhoI restriction sites.

pCS2++-DnaB I_C_-memEGFP

The membrane targeted EGFP variant was constructed by genetically engineering a membrane targeting sequence, namely c-HaRas at the C-terminus of EGFP, via sequential PCR. Initially, a PCR fragment was encoding EGFP and half of the cHaRas membrane targeting sequence was amplified using the pEGFP-N3 plasmid (from Addgene) as template. The primers used for the first PCR were: F_EGFP _(5' AGCGAATTCATGGTGAGCAAGGGCGAGGAG 3') and RA_EGFP-cHaras _(5' gggccactctcatcaggagggttcagcttCTTGTACAGCTCGTCCATGCCG 3'). A second PCR followed using this PCR product and the following primers: F_EGFP _(5' AGCGAATTCATGGTGAGCAAGGGCGAGGAG 3') and RB_EGFP-cHaras _(5' GCCTCGAGtcaggagagcacacacttgcagctcatgcagccggggccactctc 3'). This PCR fragment encoded the fusion EGFP-cHaRas and was inserted into the multiple cloning site of the pCS2++ plasmid by restriction enzyme digest with EcoRI-XhoI. A PCR fragment amplified with F_IC _(ACATCGATatgttatcaccagaaatagaaaagttgtctcag) and R_IC _(CTGAATTCgttatggacaatgatgtcattggcgac) encoding DnaB I_C _using the pMAL plasmid (kind gift from Dr. Xiang-Qin Liu) as template, was inserted upstream and in frame with the EGFP-cHaRas on pCS2++ between the ClaI-EcoRI restriction sites.

All plasmids were transcribed into RNA using mMessage mMachine Sp6 kit (Ambion) and the mRNAs were purified using the Mega Clear kit (Ambion). Microinjections performed in Ficoll as mentioned above.

### Electrophoretic analysis of protein trans-splicing

Biochemical analysis of protein-trans splicing was performed by lysis of injected *Xenopus *embryos at stage 10. Lysis was performed by pipetting up and down in the presence of proteinase inhibitors (Sigma) and DNAse (Roche). Lysates were then loaded onto agarose gels run at 100 V for 2 h, at 4°C. Gels were visualized with a UVP Imager.

### Activin-induced Cell migration assays

Animal cap explants were prepared from stage 8 embryos. Cells were dissociated in CMFM (Ca^2+ ^and Mg^2+ ^free medium) and then treated with activin protein (1 U/ml in 1×CMFM) for 1 hour. The dissociated cells were subsequently plated in Modified Barth's Solution [60] into fibronectin-coated chambered coverslips (VWR). Coverslips were coated with 0.1 mg/ml fibronectin (Sigma, diluted to the appropriate concentration with MBS) for 2 hours at room temperature, and then blocked with bovine serum albumin (BSA; 50 mg/ml in MBS).

### Image analysis

Timelapse analysis of dissociated cells was performed using a Zeiss Axiocam MR3 camera attached to a Zeiss Axiovert 135. Images were acquired and timelapse files assembled using Axiovision software 4.6.

## Competing interests

The authors declare that they have no competing interests.

## Authors' contributions

PS conceived of the study, participated in its design and coordination and helped to draft the manuscript. AC participated in the design and coordination of the study and drafted the manuscript. IA carried out carried out the molecular and biochemical studies and the in vivo assays. NC helped to carry out some of the in vivo experiments. All authors read and approved the final manuscript.
